# New evidence for the presence of the telomere motif (TTAGG) _n_ in the family Reduviidae and its absence in the families Nabidae and Miridae (Hemiptera, Cimicomorpha)

**DOI:** 10.3897/CompCytogen.v13i3.36676

**Published:** 2019-09-20

**Authors:** Snejana Grozeva, Boris A. Anokhin, Nikolay Simov, Valentina G. Kuznetsova

**Affiliations:** 1 Cytotaxonomy and Evolution Research Group, Institute of Biodiversity and Ecosystem Research, Bulgarian Academy of Sciences, Sofia 1000, 1 Tsar Osvoboditel, Bulgaria Institute of Biodiversity and Ecosystem Research, Bulgarian Academy of Sciences Sofia Bulgaria; 2 Department of Karyosystematics, Zoological Institute, Russian Academy of Sciences, St. Petersburg 199034, Universitetskaya nab., 1, Russia Zoological Institute, Russian Academy of Sciences St. Petersburg Russia; 3 National Museum of Natural History, Bulgarian Academy of Sciences, Sofia 1000, 1 Tsar Osvoboditel, Bulgaria National Museum of Natural History, Bulgarian Academy of Sciences Sofia Bulgaria

**Keywords:** Heteroptera, *Rhynocorispunctiventris*, *R.iracundus*, *Nabissareptanus*, *Horistusorientalis*, TTAGG-FISH

## Abstract

Male karyotype and meiosis in four true bug species belonging to the families Reduviidae, Nabidae, and Miridae (Cimicomorpha) were studied for the first time using Giemsa staining and FISH with 18S ribosomal DNA and telomeric (TTAGG)_n_ probes. We found that *Rhynocorispunctiventris* (Herrich-Schäffer, 1846) and *R.iracundus* (Poda, 1761) (Reduviidae: Harpactorinae) had 2n = 28 (24 + X_1_X_2_X_3_Y), whereas *Nabissareptanus* Dohrn, 1862 (Nabidae) and *Horistusorientalis* (Gmelin, 1790) (Miridae) had 2n = 34 (32 + XY) and 2n = 32 (30 + XY), respectively. FISH for 18S rDNA revealed hybridization signals on a sex chromosome, the X or the Y, in *H.orientalis*, on both X and Y chromosomes in *N.sareptanus*, and on two of the four sex chromosomes, Y and one of the Xs, in both species of *Rhynocoris* Hahn, 1834. The results of FISH with telomeric probes support with confidence the absence of the “insect” telomere motif (TTAGG)_n_ in the families Nabidae and Miridae and its presence in both species of genus *Rhynocoris* of the Reduviidae, considered as a basal family of Cimicomorpha. Increasing evidence reinforces the hypothesis of the loss of the canonical “insect” telomere motif (TTAGG)_n_ by at least four cimicomorphan families, Nabidae, Miridae, Tingidae, and Cimicidae, for which data are currently available.

## Introduction

The true bugs (Hemiptera: Heteroptera), with almost 45,000 described species distributed into 91 families and seven infraorders (Henry 2017), are one of the largest and most diverse groups of non-holometabolous insects. Overall, 40 species, 27 genera and 10 families have been studied in respect to the telomere structure (Okazaki et al. 1993, Sahara et al. 1999, Grozeva et al. 2011, Golub et al. 2015, 2017, 2018, Pita et al. 2016, Chirino et al. 2017, Angus et al. 2017). The species studied belong to three largest infraorders, including a more basal infraorder Nepomorpha and the evolutionary derived sister infraorders Pentatomomorpha and Cimicomorpha. The “insect” telomere motif (TTAGG)_n_ was found in all studied species of the families Belostomatidae (Kuznetsova et al. 2012, Chirino et al. 2017) and Nepidae (Angus et al. 2017) from the Nepomorpha. Likewise, this motif was reported for the suborder Coleorrhyncha, a sister group to the Heteroptera (Kuznetsova et al. 2015). These facts indicate that it is most likely the ancestral telomeric repeat sequence of the Heteroptera in general. In contrast, all studied species of the families Lygaeidae s.l., Pentatomidae, and Pyrrhocoridae from the Pentatomomorpha, as well as those of the families Nabidae, Tingidae, Cimicidae, and Miridae from the Cimicomorpha were shown to lack this motif (Okazaki et al. 1993, Sahara et al. 1999, Grozeva et al. 2011, Golub et al. 2015, 2017, 2018). Based on this evidence, a hypothesis was advanced that the ancestral telomeric repeat TTAGG was lost at the base of the clade Pentatomomorpha + Cimicomorpha (= the Geocorisae sensu Schuh et al. 2009) being secondarily replaced by another yet unknown motif or an alternative telomerase-independent mechanism of telomere maintenance (Frydrychová et al. 2004, Mason et al. 2016). However, a recent research of Pita et al. (2016) discovered the putative ancestral “insect” motif in the cimicomorphan family Reduviidae (the assassin bugs), namely in the comparatively young (24–38 Ma, after Hwang and Weirauch 2012) hematophagous subfamily Triatominae. Due to this finding, the validity of the above hypothesis was questioned. Moreover, the postulated lack of the (TTAGG)_n_ detection, at least in the families of Cimicomorpha, was suggested to be “due to a methodological problem of the telomeric probe rather than a loss process during their evolution” (Pita et al. 2016).

Primarily to address this issue, we did a (TTAGG)_n_FISH experiment involving four species of the Cimicomorpha, which have not previously been studied in respect to telomere composition. These are Nabis (Halonabis) sareptanus Dohrn, 1862 from the family Nabidae; *Horistusorientalis* (Gmelin, 1790) from the family Miridae; *Rhynocorispunctiventris* (Herrich-Schäffer, 1846) and *R.iracundus* (Poda, 1761) from the family Reduviidae, the subfamily Harpactorinae. Specifically, we looked for a strong evidence of the absence of the (TTAGG)_n_ telomere motif in Nabidae and Miridae as well as an additional evidence of the presence of this motif in the family Reduviidae.

In addition, we aimed to detect the 18S rDNA loci in the above species. Finally, we characterized, for the first time, the karyotype and meiotic pattern of spermatogenesis in each of the species under study.

## Material and methods

### Taxon sampling, fixation and slide preparation

The true bug specimens were collected in May-June 2018, in Bulgaria. The localities from which the bugs were collected and the number of males and mitotic/meiotic preparations studied are given in Table 1. The insects were brought to the lab and fixed alive in a fixative consisting of 3 parts of 95% ethanol and 1 part of glacial acetic acid. Chromosome preparations were made from the male gonads. The testes were extracted from the abdomen, placed on a slide in a drop of 45% acetic acid, and squashed. The coverslips were removed with a razor blade after freezing with dry ice, and the slides were, then, dehydrated in fresh fixative (3 : 1) and air dried.

**Table 1.** Material studied.

Species	Locality	Date of collection	Number of males/ preparations analysed by Shiff-Giemsa staining	Number of males/ preparations analysed by FISH
*Rhynocorispunctiventris* (Herrich-Schäffer, 1846)	Bulgaria, Kresna Gorge 41.762378N, 23.169228E	23 May 2018	1/6	1/3
*Rhynocorisiracundus* (Poda, 1761)		23 May 2018	3/11	2/5
*Horistusorientalis* (Gmelin, 1790)		23 May 2018	1/1	1/2
Nabis (Halonabis) sareptanus Dohrn, 1862	Bulgaria, Pomorie Lake 42.565609N, 27.630627E	07 June 2018	3/5	1/1

### Routine staining

For this staining, we followed the Schiff-Giemsa method described by Grozeva and Nokkala (1996).

### Fluorescence in situ hybridization (FISH)

Probes for 18S rDNA and (TTAGG)_n_ were prepared and FISH was performed according to Grozeva et al. (2015) with some modifications. For primer information, see Grozeva et al. (2011). The telomere probe (TTAGG)_n_ was amplified by PCR and labelled with rhodamine-5-dUTP (GeneCraft, Köln, Germany). An initial denaturation period of 3 min at 94 °C was followed by 30 cycles of 45 s at 94 °C, annealing for 30 s at 50 °C and extension for 50 s at 72 °C, with a final extension step of 3 min at 72 °C. The 18S rDNA probe was amplified by PCR and labelled with biotin-11-dUTP (Fermentas, Vilnius, Lithuania) using genomic DNA of the true bug *Pyrrhocorisapterus* (Linnaeus, 1758). An initial denaturation period of 3 min at 94 °C was followed by 33 cycles of 30 s at 94 °C, annealing for 30 s at 50 °C and extension for 1.5 min at 72 °C, with a final extension step of 3 min at 72 °C. The chromosome preparations were treated with 100 μg/ml RNase A and 5 mg/ml pepsin solution to remove excess RNA and proteins. Chromosomes were denatured in the hybridization mixture containing labelled 18S rDNA and (TTAGG)_n_ probes (80–100 ng per slide) with an addition of salmon sperm blocking reagent and then hybridized for 42 h at 37 °C. 18S rDNA probes were detected with NeutrAvidin-Fluorescein conjugate (Invitrogen, Karlsbad, CA, USA). The chromosomes were mounted in an antifade medium (ProLong Gold antifade reagent with DAPI, Invitrogen) and covered with a glass coverslip. The number of males involved in the study ranged from three to one (Table 1), and the number of preparations examined ranged from one (*Horistusorientalis*) to 11 (*Rhynocorisiracundus*) and the number of prophase/metaphase plates examined ranged from a few (*H.orientalis*) to several dozen (*N.sareptanus* and *Rhynocoris* spp).

As a control for the efficacy of our (TTAGG)_n_FISH experiments, we used chromosome preparations from *Scarlupelladiscolor* (Germar, 1821) (Hemiptera: Auchenorrhyncha) known to be (TTAGG)_n_ -positive (Maryańska-Nadachowska et al. 2016).

### Microscopy and imaging

The routinely stained preparations were analysed under a light microscope (Axio Scope A1 – Carl Zeiss Microscope) at 100× magnification and documented with a ProgRes MF Cool, Jenoptic (Jena, Germany). FISH images were taken using a Leica DM 6000 B microscope with a 100× objective, Leica DFC 345 FX camera, and Leica Application Suite 3.7 software with an Image Overlay module (Leica Microsystems, Wetzlar, Germany). The filter sets applied were A, L5 and N21 (Leica Microsystems). The specimens from which the chromosome preparations have been obtained are stored at the Institute of Biodiversity and Ecosystem Research, BAS (Sofia, Bulgaria).

## Results and discussion

### Family Reduviidae

**Subfamily Harpactorinae**

**Tribe Harpactorini**

**Figures 1–7.**
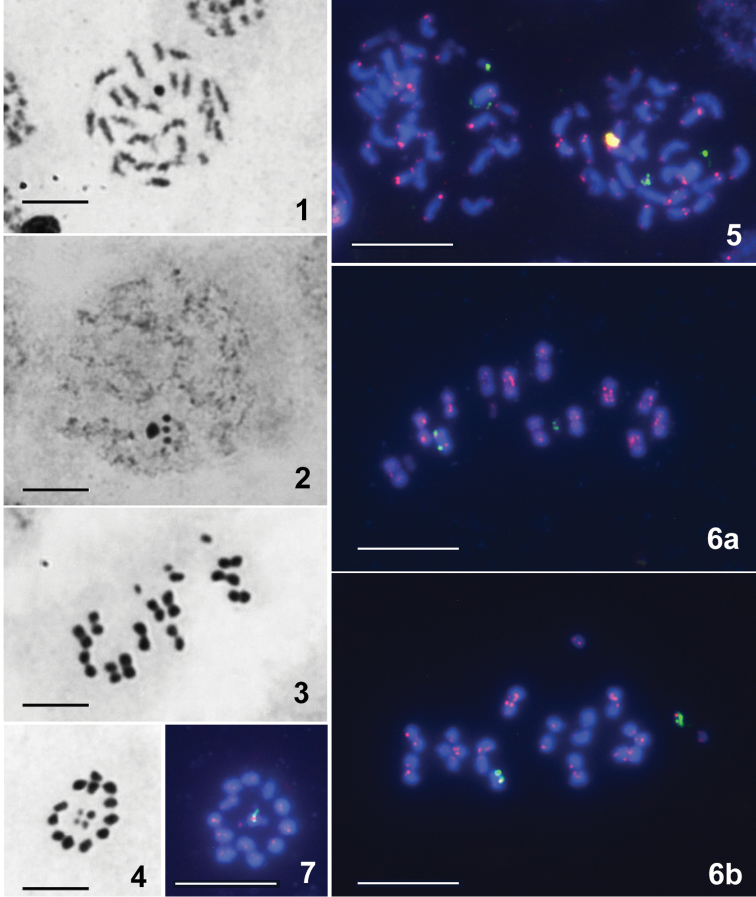
*Rhynocorispunctiventris* (**1, 3, 4, 6b, 7**) and *R.iracundus* (**2, 5, 6a**), 2n (♂) = 28 (24A + X_1_X_2_X_3_Y). Routine staining (**1–4**), FISH with 18S rDNA (green) and telomeric (TTAGG)_n_ (pink) probes (**5–7**). **1, 5** spermatogonial metaphase **2** early condensation stage with four sex chromosome bodies **3, 6a, b** metaphase I (MI) **4, 7** metaphase II (MII) with four sex chromosomes located in the center of the ring formed by autosomes. Hybridization signals of the (TTAGG)_n_ probe are seen at the ends of chromosomes, and the signals of 18S rDNA FISH are seen on the Y chromosome and on one of the X chromosomes in both species (**5–7**). Scale bars: 10 μm.

*Rhynocorispunctiventris*, 2n ♂ = 28 (24A + X_1_X_2_X_3_Y), Figs 1, 3, 4, 6b, 7

*R.iracundus*, 2n ♂ = 28 (24A + X_1_X_2_X_3_Y), Figs 2, 5, 6a

Both species were found to have 28 chromosomes at spermatogonial metaphases (Figs 1, 5), and 12 autosomal bivalents and 4 univalent sex chromosomes at spermatocyte metaphases I (MI) (Figs 3, 6a, b). Such a chromosomal complement has been reported for all so far studied species of the genus *Rhynocoris* Hahn, 1834 and also for half the studied species of the tribe Harpactorini (see for review: Tiepo et al. 2016). The autosomes of spermatogonial metaphases and in turn both bivalents (MI) and univalent autosomes (MII) in meiosis are of a more or less similar size. Among the four sex chromosomes, the largest is considered as the Y and the others as X_1_, X_2_, and X_3_ that could have originated through the fission processes of the original X chromosome of an ancestor with a simple system XY. Although we studied no females and have thus no direct confirmation of such interpretation of sex chromosome system in these two species, it is likely, as it represents the prevalent pattern reported for their close relatives (Tiepo et al. 2016). Three X chromosomes are of similar size and the smallest chromosomes of the complement. At condensation stage of meiosis, the four sex chromosome bodies were observed (Fig. 2). The analysis of MI and MII plates confirmed that the sex chromosomes followed the conventional in the Heteroptera (Ueshima 1979) post-reductional mode of separation of the sex chromosomes, i.e., they divide in the first division and segregate in the second division. As with other reduviid species (see e.g. Poggio 2007, Tiepo et al. 2016), at MII the autosomes are arranged to form a ring, with sex chromosomes being positioned inside the ring as a pseudo-tetravalent without having a visible connection between them (Figs 4, 7).

**Figure 8.**
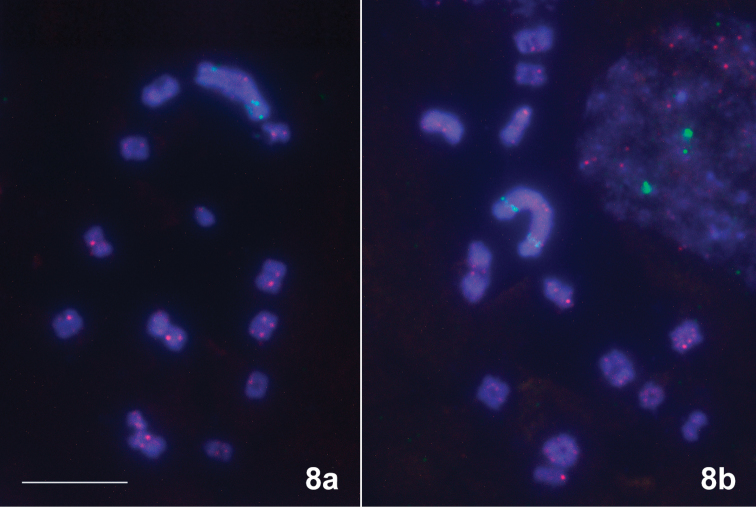
*Scarlupelladiscolor*. FISH with 18S rDNA (green) and telomeric (TTAGG)_n_ (pink) probes. Hybridization signals of the (TTAGG)_n_ probe are seen at the ends of chromosomes. Scale bar: 10 μm.

Figures 5 to 7 present the results of the application of FISH with (TTAGG)_n_ and 18S rDNA probes to mitotic and meiotic chromosomes of *R.iracundus* (Figs 5, 6a) and *R.punctiventris* (Figs 6b, 7). Hybridization signals of the telomeric probe are clearly seen on the ends of chromosomes of both species indicating that their telomeres contain the canonical insect telomeric TTAGG tandem repeat. However not all chromosome ends show bright hybridization signals. The same variation in both the number and/or the intensity of signals has repeatedly been described in other true bug species (Pita et al. 2016, Angus et al. 2017, Chirino et al. 2017). Moreover, it was also observed at MI plates from *Scarlupelladiscolor* (Auchenorrhyncha) used here as a positive control for the (TTAGG)_n_ probe in all our FISH experiments (Fig. 8a, b). Such variation may be due to differences in the length of target TTAGG sequences (Chirino et al. 2017) or uneven access of the probe to the chromosomes (Pita et al. 2016). In all the figures presented, 18S rDNA FISH signals are seen on sex chromosomes, the Y and one of the X chromosomes. The condensation of sex chromosomes at MI made it impossible to determine the precise location of rDNA sites on them. At MI, these chromosomes are split into the sister chromatids and consequently show each twin hybridization signals of both telomeric and rDNA probes (Figs 6a, b). A similar pattern of the rDNA distribution was previously reported for *Cosmoclopiusnigroannulatus* (Stål, 1860), another Harpactorini species with the same karyotype 2n = 24A + X_1_X_2_X_3_Y (Bardella et al. 2014).

### Family Nabidae

**Subfamily Nabinae**

**Tribe Nabini**

**Figures 9–14.**
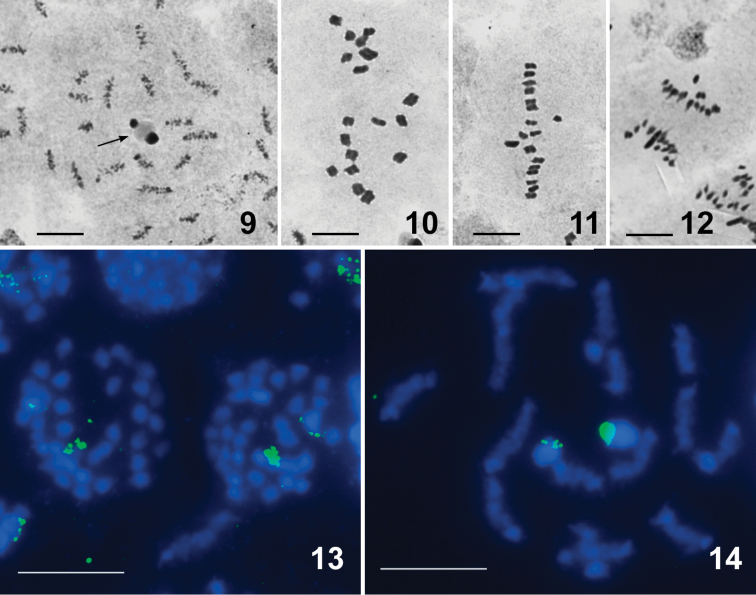
Nabis (Halonabis) sareptanus, 2n (♂) = 34 (32A + XY). Routine staining (**9–12**), FISH with 18S rDNA (green) and telomeric (TTAGG)_n_ (pink) probes. (**13, 14**); **9, 14** condensation stage (**9** at the early condensation stage, 2 univalent sex chromosomes are positively heteropycnotic and associate to one another via a nucleolus; arrowed) **10**MI**11**MII**12** anaphase II (AII) **13** spermatogonial metaphase. There are no hybridization signals of the (TTAGG)_n_ probe; the signals of the 18S rDNA probe are seen on both X and Y chromosomes (**13, 14**). Scale bars: 10 μm.

Nabis (Halonabis) sareptanus, 2n ♂ = 34 (32A + XY), Figs 9–14

The chromosome complement of males studied here agrees with that reported earlier for males of this species originating from the Republic of Kazakhstan (Kuznetsova and Maryańska-Nadachowska 2000). However, the cited paper provided neither descriptions nor illustrations of karyotype and meiosis. According to our observations of different stages of meiosis, the autosomes of this species more or less gradually decrease in size; the X far exceeds in size the largest autosome, whereas the Y is one of the medium-sized elements of the complement (Figs 9–14). At the condensation stage, there are 16 autosomal bivalents and 2 univalent sex chromosomes, which are positively heteropycnotic and associate to one another via a nucleolus (Fig. 9). The first division is reductional for the autosomes and equational for the sex chromosomes (sex chromosome post-reduction). Figures 9 and 14 present late condensation stages with 16 autosomal bivalents and univalent chromosomes X and Y, which split into the sister chromatids each. As in other nabid species (Nokkala and Nokkala 1984, Kuznetsova and Maryańska-Nadachowska 2000), the homologues of every bivalent align in parallel without chiasmata between them (Figs 10, 14), i.e. meiosis is achiasmate of the so-called *alignment* type (Nokkala and Nokkala 1984). During the second division, sex chromosomes show “distance pairing” at MII (Fig. 11) and move to different poles at anaphase II (Fig. 12).

FISH with the (TTAGG)_n_ probe revealed no signals on chromosomal spreads of *N.sareptanus* (Figs 13, 14) suggesting thus that its telomeres lack the “insect” telomere motif (TTAGG)_n_. The hybridization signals of the 18S rDNA probe, as expected because of the association of the nucleolus with the sex chromosomes (see above), were present on both X and Y sex chromosomes (Figs 13, 14). This is the first evidence of the rDNA location in the family Nabidae.

### Family Miridae

**Subfamily Mirinae**

**Tribe Mirini**

**Figure 15.**
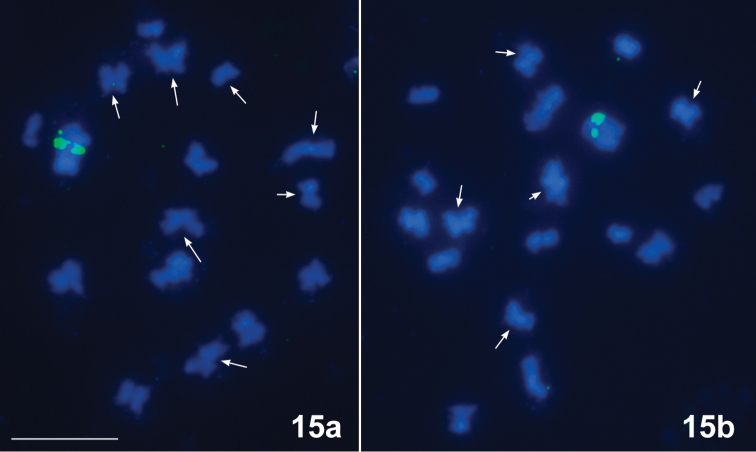
*Horistusorientalis*, 2n (♂) = 32 (30A + XY). FISH with 18S rDNA (green) and telomeric (TTAGG)_n_ (pink) probes. Early MI – bivalents with one or occasionally two tenacious threads, the so-called *collochores* (arrowed). There are no hybridization signals of the (TTAGG)_n_ probe; the signals of the 18S rDNA probe are seen on the XY sex chromosome pseudo-bivalent. Scale bar: 10 μm.

*Horistusorientalis*, 2n ♂ = 32 (30A + XY), Fig. 15

Fifteen bivalents of autosomes and a pseudo-bivalent composed of the X and Y sex chromosomes are present at early MI (Fig. 15a, b). No chiasmata are present in the bivalents; however, one or occasionally two tenacious threads, the so-called *collochores*, hold the homologues together. This pattern, known as the *collochore* type of achiasmate meiosis (Nokkala and Nokkala 1986) was described in all hitherto studied representatives of the family Miridae (for references see Kuznetsova et al. 2011).

FISH with the (TTAGG)_n_ probe revealed no signals on chromosomal spreads of *H.orientalis* suggesting thus that its telomeres lack, as in *N.sareptanus*, the “insect” telomere motif (TTAGG)_n_. The twin hybridization signals of the 18S rDNA probe were seen on the XY sex chromosome pseudo-bivalent; however, we failed to understand whether they were present on the X or on the Y chromosome. In two another species of the family Miridae studied previously in this respect, *Deraeocorisrutilus* (Herrich-Schaeffer, 1838) and *D.ruber* Linnaeus, 1758, both with an XY sex chromosome system, rDNA clusters were shown to be located on the X chromosome and on both X and Y chromosomes, respectively (Grozeva et al. 2011).

## Conclusion

The major result of our work is a compelling support for the absence of the canonical “insect” telomeric TTAGG tandem repeat in the families Nabidae and Miridae (Table 2). It now seems clear that prior notions of these families as groups lacking the insect telomere motif (TTAGG)_n_ (Frydrychová et al. 2004, Grozeva et al. 2011) are correct. As mentioned in the Introduction, this motif was also not discovered in two another cimicomorphan families, namely, Cimicidae and Tingidae (Grozeva et al. 2004, Golub et al. 2015, 2017, 2018) and in all so far studied species of the sister to the Cimicomorpha infraorder Pentatomomorpha (in the families Lygaeidae s.l., Pyrrhocoridae, and Pentatomidae) (Frydrychová et al. 2004, Grozeva et al. 2011). Mason et al. (2016) have suggested a single loss event of the TTAGG telomeric repeat before the Cimicomorpha and Pentatomomorpha divergence, and after their separation from the Nepomorpha. The discovery of this motif in the supposedly monophyletic family Reduviidae, both in the second largest subfamily Triatominae (Pita et al. 2016) and in the largest subfamily Harpactorinae (present study), allows diverse speculations.

**Table 2.** Karyotypes and results of FISH mapping of telomere (TTAGG)_n_ motif and 18S rDNA loci.

Taxon	2n ♂	Presence / absence of (TTAGG)_n_ motif	Location of 18S rDNA loci
**Family Reduviidae**			
*Rhynocorispunctiventris*	28 (24A + X_1_X_2_X_3_Y)	Present	Y and one of the X chromosomes
*Rhynocorisiracundus*	28 (24A + X_1_X_2_X_3_Y)	Present	Y and one of the X chromosomes
**Family Miridae**			
*Horistusorientalis*	32 (30A + XY)	Absent	One of the sex chromosomes (unidentified)
**Family Nabidae**			
Nabis (Halonabis) sareptanus	34 (32A + XY)	Absent	Both X and Y chromosomes

With approximately 6,800 described species in 25 subfamilies, the assassin bugs represent one of the largest families within the order Hemiptera. Phylogeny and relationships within and between subfamilies of the Reduviidae are far from being resolved (Hwang and Weirauch 2012). According to most available phylogenies, Reduvioidea (Reduviidae + Pachynomidae) are monophyletic and a sister group to the rest Cimicomorpha (Schuh et al. 2009, Weirauch and Schuh 2011). We can therefore assume a scenario where an ancestor of Cimicomorpha + Pentatomomorpha possessed the ancestral in Insecta (Frydrychová et al. 2004) and most likely initial in the Heteroptera (Kuznetsova et al. 2012) motif (TTAGG)_n_, that retained in the Reduviidae but was then repeatedly lost by other families of the Geocorisae. Similarly, the huge insect orders Coleoptera and Hymenoptera include both TTAGG-positive and TTAGG-negative species, which was interpreted as the multiple loss of the initial telomeric sequence during their evolution (Frydrychová and Marec 2002, Gokhman et al. 2014, Menezes et al. 2017). To be sure, there remains much work toward elucidating the problem and testing the above hypothesis. Future telomere TTAGG-FISH analyses should focus on including additional species and higher-level taxa of the Heteroptera.
